# Feasibility of a digital therapeutic for experiential negative symptoms of schizophrenia: results from an exploratory study

**DOI:** 10.1038/s41537-025-00659-1

**Published:** 2025-09-26

**Authors:** Haig Goenjian, Abhishek Pratap, Cassandra Snipes, Brendan D. Hare, Joshua T. Kantrowitz, Tshekedi Dennis, Wakelin McNeel, Eehwa Ung, Olya Besedina, Alankar Gupta, Tim Campellone, Mariya Petrova, Sergio Perocco, Uma Vaidyanathan, Shaheen E. Lakhan, Cornelia Dorner-Ciossek

**Affiliations:** 1 CenExel CNS, Garden Grove and Los Alamitos, Los Alamitos, CA USA; 2 Boehringer Ingelheim Pharmaceuticals, Inc, Ridgefield, CT USA; 3 Click Therapeutics, Inc., New York, NY USA; 4 Columbia University Department of Psychiatry and New York State Psychiatric Institute (NYSPI), New York, NY USA; 5 Velocity Clinical Research, Santa Ana, CA USA; 6 BHC Alhambra Hospital, Rosemead, CA USA; 7 Boehringer Ingelheim International GmbH, Ingelheim am Rhein, Germany

**Keywords:** Schizophrenia, Human behaviour

## Abstract

Experiential negative symptoms (ENS) of schizophrenia, such as asociality, anhedonia, and avolition, are associated with poor outcomes, yet no FDA-approved pharmacotherapies currently exist specifically to target these symptoms. With the increasing use of smartphones, evidence-based digital interventions delivered by prescription digital therapeutics (DTx) may present an opportunity to address the unmet therapeutic need for ENS of schizophrenia. CT‑155/BI 3972080 (CT-155) is being developed as a smartphone-based prescription DTx for the treatment of ENS. A multicenter, 7-week, single-arm, open-label, exploratory study (NCT05486312) evaluated the engagement, adherence, potential effectiveness, acceptability, user experience, and safety of an abbreviated version of CT-155 (CT‑155 beta). Engagement and adherence with CT-155 beta were measured passively throughout the study using the study app. Change in ENS severity was assessed using the clinically administered clinical assessment interview for negative symptoms, motivation, and pleasure subscale (CAINS-MAP). Acceptability and user experience were assessed using the validated Mobile App Rating Scale (MARS) along with an episodic user experience survey, respectively. Fifty participants with a clinically confirmed schizophrenia diagnosis were enrolled; 80% were male, 58% were Black or African American, and the median (range) age was 53.5 (23–64) years. At baseline, participants' mean (SD) CAINS-MAP total score was 20.5 (8.3). Most participants (*n* = 43; 86%) completed the 7-week study. Participants readily engaged with CT-155 beta. Kaplan–Meier retention analysis showed that 84% of participants (*N* = 42/50) engaged with CT-155 beta (i.e., last app open) until the end of the study period. Daily app check-ins were completed on a median (IQR) of 43.0 (19–47) days of the 49 possible days (88%). The median (IQR) duration of engagement was 11.6 (8.1–16.1) min per session. Additionally, adherence with CT-155 was high, with participants completing a median of 18 (IQR 13–20) of the 21 therapeutic lessons available. After 7 weeks of CT-155 beta usage, the mean change in within-subject CAINS-MAP score was 3.6 points from baseline (95% CI 1.3, 5.8; *p* = 0.0026; baseline: 20.4 (8.6) Week 7: 16.8 (7.7)). Most participants (91%; *n* = 39/43) rated CT-155 beta functionality using MARS assessment as acceptable or higher, with an overall mean MARS functionality subscale score of 4.2 points out of 5 points, with 5 corresponding to “excellent” at Week 7. The end of study participant feedback survey showed that 95% (*n* = 42/44) of participants would recommend using CT-155 beta. No app-related adverse events nor severe adverse events leading to discontinuation of the study were reported. Overall, the study demonstrated the feasibility of CT-155 beta in participants with ENS of schizophrenia. Results from this feasibility study show the potential of evidence-based DTx approaches to address ENS of schizophrenia.

## Introduction

Schizophrenia is a complex and disabling psychiatric disorder that affects approximately 0.45% of adults worldwide^1^ and 0.25–64% of adults in the US^2–4^, imposing a substantial health, social, and economic burden^5,6^. Furthermore, schizophrenia disproportionately impacts marginalized communities, a pattern attributed not to increased biological vulnerability but to a combination of social determinants, structural inequalities, and potential diagnostic bias^7,8^. Symptoms of schizophrenia are described in terms of positive, negative, and cognitive symptoms, and 50–60% of people living with schizophrenia experience at least one negative symptom^9,10^. Although antipsychotics have been shown to reduce positive symptoms (i.e., delusions and hallucinations) and can also improve secondary negative symptoms^11^ (e.g., blunted affect arising from the emotional distress of delusions or hallucinations), enduring primary negative symptoms typically do not respond to currently available antipsychotics^12^. While several antipsychotics are approved by the Food and Drug Administration (FDA) for the treatment of schizophrenia, none are specifically approved to target cognitive and negative symptoms, which remain inadequately addressed by current pharmacological options^13,14^.

Negative symptoms can be subdivided into expressive negative symptoms (blunted affect and alogia)^15–18^, and experiential negative symptoms (ENS; avolition, anhedonia, and asociality)^15,16,18,19^. ENS have been associated with poorer outcomes than expressive negative symptoms, including significantly greater conceptual disorganization and psychosis, increased likelihood of hospitalization, poorer social functioning, greater social cognitive impairment, greater likelihood of unemployment^20^, fewer hours worked, and a reduction in wages earned^20,21^. Furthermore, people with schizophrenia who have elevated ENS (i.e., those with higher asociality, avolition, and/or anhedonia) report reduced quality of life compared to those with less severe ENS^22^.

Individuals with negative symptoms often exhibit impaired positive reward learning and heightened defeatist performance beliefs^23,24^. Research has shown that engaging individuals in rewarding activities and value-based goal setting has shown significant reductions in avolition-apathy negative symptoms and improvement in global functioning vs treatment as usual^25–27^. Furthermore, cognitive behavioral therapy (CBT), along with other psychosocial treatments including social skills training and cognitive remediation, are recommended in clinical guidelines as effective strategies to augment other treatments, and may all improve ENS^12,28–32^. While psychosocial approaches have proven effective in managing negative symptoms, their accessibility and uptake can be limited due to insufficient resources (e.g., a lack of trained professionals to deliver these interventions) and poor healthcare resource utilization in general^33,34^. Furthermore, negative symptoms often predict poor engagement with clinical services, creating an additional barrier to treatment^35^. These barriers to psychosocial accessibility and uptake, as well as the substantial burden of ENS on people living with schizophrenia, highlight the need for new, effective, and accessible therapies. These new treatments could significantly enhance current care options and improve the overall continuum of support for patients, ultimately leading to better health outcomes and quality of life.

Digital therapeutics (DTx), defined by the International Organisation for Standardization as “health software intended to treat or alleviate a disease, disorder, condition, or that has a demonstrable positive therapeutic impact on a patient’s health,”^36^ may address this unmet need. DTx can be delivered remotely to augment existing mental health services in a way that is flexible and convenient for patients, and therefore has the potential to broaden access to evidence-based psychosocial therapies^37,38^. Indeed, DTx has demonstrated effectiveness in difficult-to-treat populations, including people with post-traumatic stress disorder^39,40^, substance use disorder^41,42^, and attention deficit hyperactivity disorder^43,44^. Although DTx for people living with schizophrenia is more limited, promising results have emerged^45–49^. For example, a study demonstrated significant reductions in ENS and defeatist beliefs in a cohort of 31 patients with psychosis after 12 weeks of mobile-assisted CBT^48^. In another study, a DTx designed to improve motivational impairments in patients with early psychosis led to significant improvements in defeatist beliefs, motivation, and self-efficacy^49^.

CT-155/BI 3972080 (CT-155), is being developed as a smartphone-based prescription DTx that provides psychosocial intervention techniques adjunctive to standard of care treatments for patients 18 years of age and older with ENS of schizophrenia. The CT-155 program has been granted Breakthrough Device designation by the FDA^50,51^. CT-155 was designed in line with FDA recommendations that emphasize the importance of incorporating patient experience data into the development process^52^, by taking into consideration the unique characteristics and needs of the schizophrenia population and integrating feedback from individuals with lived experience of schizophrenia^53^. In this exploratory study, we examined the engagement, adherence, within-group effectiveness, acceptability, user experience, and safety of a beta version of CT-155 (CT-155 beta; an abbreviated version of CT-155/BI 3972080).

## Results

### Demographics and baseline clinical characteristics

**Fig. 1:**
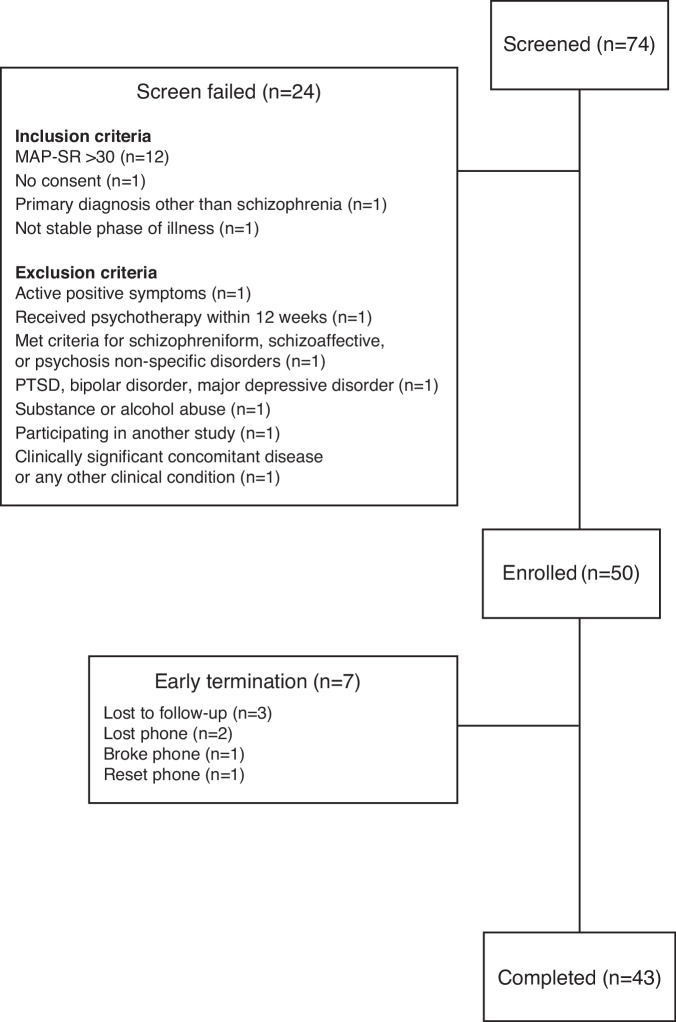
Participant disposition. PTSD post-traumatic stress disorder, MAP-SR motivation and pleasure scale—self report. Figure was adapted from Snipes et al *JMIR Mental Health* 2025 licensed under Creative Commons Attribution CC-BY 4.0^53^.

Seventy-four individuals were screened, of whom 50 were enrolled (32% screen fail rate) and 43 (86%) completed the study (Fig. 1). The median age of the cohort was 53.5 years (range: 23–64), and the majority (*n* = 40, 80%) were male. Twenty-nine (58%) participants were Black or African American, 32 (64%) participants did not have a college-level education, and 47 (94%) participants had a self-reported annual income of <$25,000. Complete sociodemographic characteristics are listed in Table 1, and clinical characteristics are listed in Table 2. The mean (standard deviation [SD]) Motivation and Pleasure scale – Self Report (MAP-SR) score at screening was 14.9 (8.9) points, and the mean Clinical Assessment Interview for Negative Symptoms Motivation and Pleasure scale (CAINS-MAP) total score at baseline was 20.5 (8.3) points. Most participants demonstrated proficiency in the use of mobile devices prior to accessing CT-155 beta, as indicated by the median (interquartile range [IQR]) total mobile device proficiency questionnaire (MDPQ) score at baseline of 30.1 (21.4, 35.5) points. Concomitant use of second-generation antipsychotics was reported by all 50 participants (*n* = 44 receiving 1 antipsychotic; *n* = 6 receiving 2 antipsychotics) (Table 2 and Supplementary Table 1).

**Table 1:** Baseline sociodemographic characteristics.

Baseline characteristic	Total (*N* = 50)
Male, *n* (%)	40 (80)
Age, median (range), years	53.5 (23–64)
Race, *n* (%)	
Black or African American	29 (58)
White	15 (30)
Asian	3 (6)
American Indian or Alaska Native	1 (2)
Other	2 (4)
Ethnicity, *n* (%)	
Hispanic or Latino	12 (24)
Not Hispanic or Latino	38 (76)
Education, *n* (%)	
Less than high school	7 (14)
High school	25 (50)
Some college	12 (24)
College degree	6 (12)
Annual income, *n* (%)	
<$25,000	47 (94)
$25,000–$49,999	3 (6)
Digital literacy	
MDPQ, mean (SD)	28.7 (8.3)

*MDPQ* mobile device proficiency questionnaire.

Table was reproduced from Snipes et al, JMIR Mental Health 2025, licensed under Creative Commons Attribution CC-BY 4.0.^53^

**Table 2:** Clinical characteristics.

Clinical characteristic	Total (*N* = 50)
Age at diagnosis, median (range), years	26 (16–52)
Time since diagnosis, median (range), years	15 (2–42)
Screening assessment	
MAP-SR, mean (SD)	14.9 (8.9)
Baseline assessments	
CAINS-MAP, mean (SD)	20.5 (8.3)
PSP^1^, mean (SD)	54.3 (12.9)
Number of concomitant antipsychotic medications used^2^	
Diazepines, oxazepines, thiazepines, and oxepines	31
Other antipsychotics	18
Indole derivatives	7

*CAINS-MAP* clinical assessment interview for negative symptoms motivation and pleasure subscale, *MAP-SR* motivation and pleasure scale—self report, *PSP* personal and social performance, *SD* standard deviation.

^1^Adjusted using clinical judgment.

^2^Concomitant use of antipsychotics was reported by all 50 participants (*n* = 44 receiving 1 antipsychotic; *n* = 6 receiving 2 antipsychotics).

### Participant engagement and adherence with CT-155 beta

Participants’ engagement with CT-155 beta was measured passively throughout the intervention period by the app. Engagement metrics included daily app opens, daily check-ins completed, and app session length. In addition, adherence metrics included the proportion of therapeutic lessons and the number of therapeutic goals completed.

**Fig. 2:**
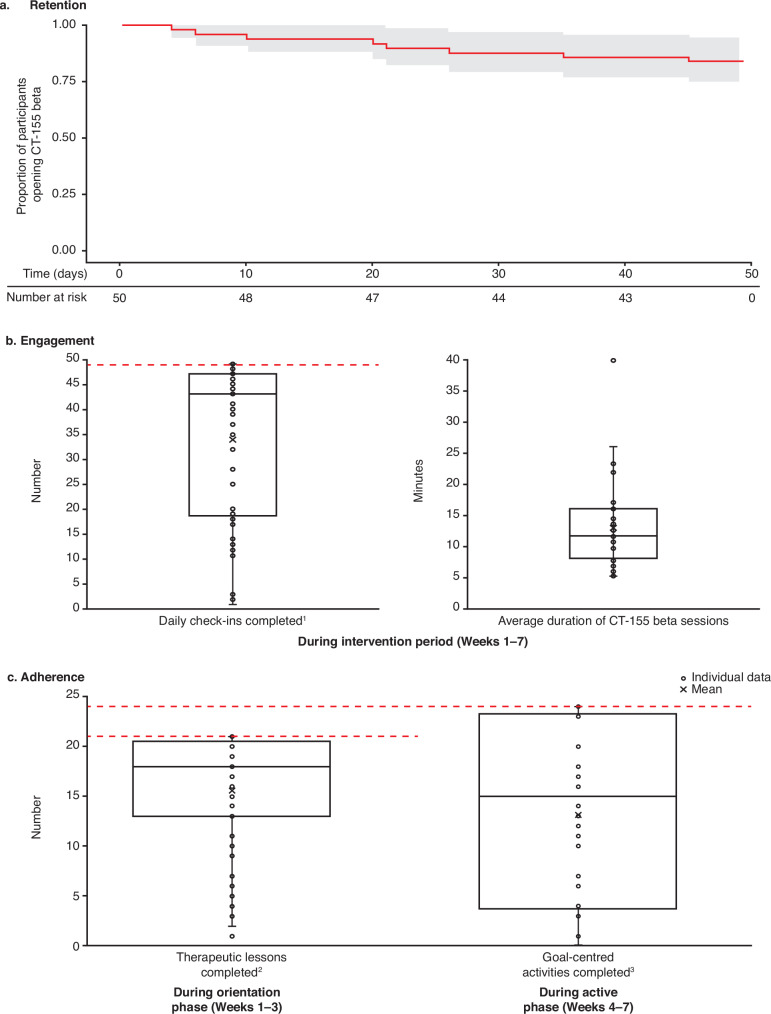
Participant engagement and adherence with CT-155 beta. **a** Kaplan–Meier curve showing participant retention (last app open) (**b**) summary of participant engagement metrics and **c** summary of adherence metrics. ^1^Forty-nine total possible days; ^2^Twenty-one total therapeutic lessons (18 required core and 3 bonus lessons); and ^3^defined as completing an activity or reporting the completion of an activity in CT-155 beta. The time (days) to when participants stopped engaging with CT-155 beta during the engagement period (*n* = 50) was analyzedusing a Kaplan–Meier survival plot. If a participant skipped some days, but returned to continue with activities, then the last day of their engagement was considered the day that they stopped engaging with CT-155 beta. Box plots depict the median and IQR of participant engagement metrics; the median is represented by a horizontal line in each box. The whiskers depict the range within 1.5 times the IQR, values outside this range are considered outliers. The red dashed line indicates the maximal attainable number where applicable.

Kaplan–Meier retention analysis shows that the majority of the participants (84%; *N* = 42/50) engaged with CT-155 beta (i.e., last app open) until the end of the study period (Day 49; Fig. 2a). The median (IQR) daily app check-ins were completed on 43.0 (19–47) days of the 49 possible days (88%) (Fig. 2b). In terms of time spent in the app, on average participants engaged with CT-155 beta for 11.6 (8.1–16.1) min per session (Fig. 2b).

Overall, there was no significant correlation between baseline CAINS-MAP score and engagement with CT-155 beta (*p* > 0.05) (Supplementary Table 2). In addition, daily app engagement with sessions of 60 s or longer was also analyzed to further characterize meaningful user engagement with the study app (Supplementary Fig. 1).

In terms of adherence to therapeutic lessons in CT-155 beta, participants completed a median (IQR) of 18 (13–20) therapeutic lessons out of the total of 21 available. Furthermore, during the active phase, the median (IQR) number of goal-centered activities completed was 15 (4–23; Fig. 2c) out of 24 available, and a median (IQR) of 4 (0–5) goals was completed per participant. This indicates on average, 1 goal per week was completed.

### Negative symptoms

**Fig. 3:**
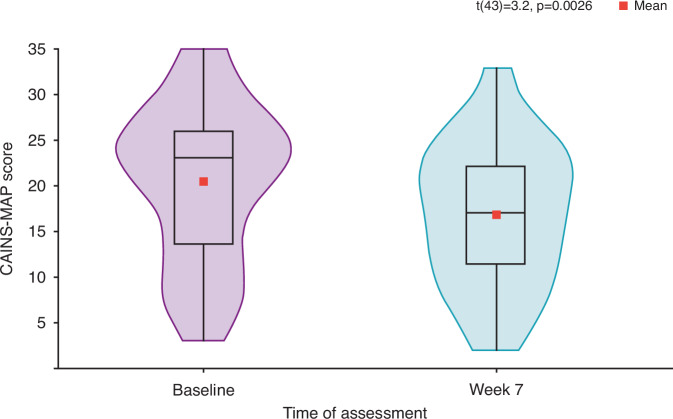
Individual and mean (violin plot) CAINS-MAP scores at baseline and Week 7^1^. ^1^Twenty-nine of the 44 participants included in the CAINS-MAP analysis were from a single study site. Within-subject changes in CAINS-MAP scores at baseline were compared with those at Week 7 using a Student’s paired *t*-test. CAINS-MAP clinical assessment interview for negative symptoms, motivation, and pleasure subscale; ENS experiential negative symptoms.

Severity of participants’ ENS of schizophrenia was evaluated by the CAINS-MAP assessment measure^15^. For those participants with baseline and Week 7 assessments (*n* = 44) baseline mean CAINS-MAP total score was 20.4 (8.6) points. At the end of the intervention period (Week 7), the mean CAINS-MAP total score was 16.8 (7.7) points (Fig. 3). This reflects an average within subject reduction of 17.6% in the mean (95% confidence interval [CI]) CAINS-MAP total score of 3.6 (1.3, 5.8) points from baseline (*t*[43] = 3.2; *p* = 0.0026; *n* = 44).

Notably, the improvement in CAINS-MAP total at Week 7 was driven by a change in two sub-domains: (i) social subscale score (the average Likert score out of 4) (median [IQR] −0.38 [–0.88, 0.00]; *p* = 0.0020) and (ii) recreational subscale score (−0.67 [−1.33, 0.00]; *p* = 0.0001). No significant change from baseline was observed in the Work & School subscale score (0.00 [−0.50, 0.50]; *p* = 0.5873) at Week 7 (Table 3).

**Table 3:** Change in CAINS-MAP subscale scores from baseline visit to Week 7.

	Median (IQR)	*p* value
Change in CAINS Social Score, *n* = 44	−0.38 (−0.88, 0.00)	0.0020^1^
Change in CAINS Work & School Score, *n* = 44	0.00 (−0.50, 0.50)	0.5873^2^
Change in CAINS Recreation Score, *n* = 44	−0.67 (−1.33, 0.00)	0.0001^1^

Data presented from participants with observed data (*n* = 44).

^1^Paired samples *t*-test.

^2^Wilcoxon signed rank test.

### Acceptability and user experience of CT-155 beta

**Fig. 4:**
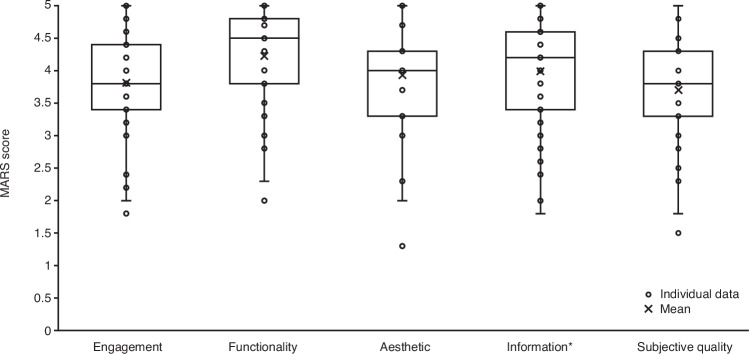
Modified^*^ MARS domain scores at Week 7^1^. ^1^*n* = 43 participants with observed data. ^*^Two items were omitted from the information domain due to a lack of relevance. Individual MARS subscale scores are shown, and ‘X’ indicates the mean. Box plots depict the median and IQR of MARS subscale scores; the median is represented by a horizontal line in each box. The whiskers depict the range within 1.5 times the IQR, values outside this range are considered outliers. MARS includes several domains: Engagement (items 1–5), functionality (items 6–9), esthetics (items 10–12), and information quality (items 14–18). Items were rated as either 1 (inadequate), 2 (poor), 3 (acceptable), 4 (good), or 5 (excellent), and statistically summarized per domain. The MARS also includes a subjective quality domain (items 20–23), which is scored separately using a 5-point Likert scale, with higher scores indicating better quality. The MARS app-specific items were scored as either 1 (strongly disagree), 2 (slightly disagree), 3 (neutral), 4 (slightly agree), or 5 (strongly agree). MARS mobile app rating scale.

Participants’ rating of study app quality and satisfaction was evaluated at the end of the study using a validated Mobile App Rating Scale (MARS) assessment. Participants rated the quality of CT-155 beta as either acceptable or good across the four key domains of the assessment—engagement, functionality, esthetics, and information quality (Fig. 4 and Supplementary Table 3). Across these four domains, the mean MARS scores ranged from 3.8 (0.8) to 4.2 (0.8) points out of 5 (“excellent”) on the Likert scale for the engagement and functionality domains, respectively. The subjective quality domain, asking about recommendation of the app, expected frequency of usage over the next 12 months, willingness to pay for the app, and star rating, was scored in the average range (mean score: 3.7 [0.8]; Fig. 4 and Supplementary Table 3). Of the 43 participants who responded, the majority of them scored the app-specific items as either 4 or 5 on the Likert scale indicating that they slightly or strongly agreed; knowledge (32/43), attitudes (29/43), awareness (29/43), intention to change (26/43), help seeking (27/43), and behavior change (26/43) (Supplementary Fig. 2 and Supplementary Table 3).

In addition, participants’ subjective experiences of using CT-155 beta were gathered through a brief user experience survey administered at Weeks 3, 5, and 7. Most participants reported using CT-155 beta either once a day or multiple times a day consistently over the 7-week intervention period (Week 3: 91.1% [41/45], Week 5: 91.1% [41/45]; Week 7: 84.1% [37/44]) and 95.5% would recommend the app to friends by the end of the study (Table 4). For free-text response questions, participants reported that CT-155 beta helped to stabilize their mood, improve their confidence, and helped them to change their perspective/outlook on life (Table 5). Overall, only a small proportion of participants provided suggestions for improving CT-155 beta, such as adding more content (~11–14%), increasing interactivity (~7%), and modifying videos (~2–7%; Table 5). Finally, feedback from the end-of-study user experience survey indicated that 89% (39/44) of participants agreed or strongly agreed that the study app was an effective treatment for their negative symptoms.

**Table 4:** User experience survey.

Question	Response	Week 3 *n* = 45	Week 5 *n* = 45	Week 7 *n* = 44
How often have you used the app?	Multiple times a day, *n* (%)	6 (13.3)	4 (8.9)	7 (15.9)
	Once a day, *n* (%)	35 (77.8)	37 (82.2)	30 (68.2)
	A few times a week, *n* (%)	1 (2.2)	2 (4.4)	4 (9.1)
	Once a week, *n* (%)	2 (4.4)	2 (4.4)	3 (6.8)
	Haven’t used it recently, *n* (%)	1 (2.2)	0 (0.0)	0 (0.0)
	Missing, *n*	5	5	6
Would you recommend this app to a friend?	Yes, *n* (%)	42 (93.3)	40 (88.9)	42 (95.5)
	No, *n* (%)	3 (6.7)	5 (11.1)	2 (4.5)
	Missing	5	5	6

*SD* standard deviation.

**Table 5:** Participants’ subjective experiences of using CT-155 beta (free text responses).

		Week 3% (*n*/*N*)	Week 5% (*n*/*N*)	Week 7% (*n*/*N*)
What are the ways the app has changed how you approach challenges in your life	New perspective/outlook on life	37.8 (17/45)	33.3 (15/45)	20.5 (9/44)
	Other—positive	20.0 (9/45)	11.1 (5/45)	18.2 (8/44)
	Mood stabilizer	17.8 (8/45)	11.1 (5/45)	15.9 (7/44)
	Relationships	6.7 (3/45)	6.7 (3/45)	9.1 (4/44)
	Confidence builder	6.7 (3/45)	13.3 (6/45)	15.9 (7/44)
	No change	4.4 (2/45)	6.7 (2/45)	6.8 (3/44)
	Improves focus and awareness	4.4 (2/45)	13.3 (6/45)	6.8 (3/44)
	Other—negative	2.2 (1/45)	0	0
Tell us about a time when you felt frustrated by your experience with the app	Never	66.7 (30/45)	64.4 (29/45)	59.1 (26/44)
	Specific exercise or situation	13.3 (6/45)	8.9 (4/45)	9.1 (4/44)
	External sources of frustration	8.9 (4/45)	8.9 (4/45)	6.8 (3/4)
	Rarely (first time or once)	4.4 (2/45)	2.2 (1/45)	2.3 (1/44)
	Technical	2.2 (1/45)	6.7 (3/45)	6.8 (3/44)
	General app-related frustration	2.2 (1/45)	4.4 (2/45)	11.4 (5/44)
	Frequently	2.2 (1/45)	4.4 (2/45)	2.3 (1/44)
	No response	0	0	2.3 (1/44)
Suggestions for improvement	No change	55.6 (25/45)	57.8 (26/45)	59.1 (26/44)
	More content	11.1 (5/45)	13.3 (6/45)	13.6 (6/44)
	Technical features	8.9 (4/45)	4.4 (2/45)	4.5 (2/44)
	Modify videos	6.7 (3/45)	4.4 (2/45)	2.3 (1/44)
	Increased interactivity	6.7 (3/45)	6.7 (3/45)	6.8 (3/44)
	Other	4.4 (2/45)	13.3 (6/45)	9.1 (4/44)
	Computer accessibility	4.4 (2/45)	0	0

### Safety

Three adverse events (AEs) were reported by 2 participants during the study, none of which were considered severe or related to CT-155 beta, nor led to discontinuation of the study. AEs reported were a sinus infection (*n* = 1), arthralgia (*n* = 1), and a rash (*n* = 1).

## Discussion

This exploratory study aimed to evaluate the feasibility of an investigational digital therapeutic app (CT-155 beta) as a potential treatment of ENS of schizophrenia, in terms of engagement, adherence, effectiveness, acceptability, user experience, and safety. The study shows that participants living with schizophrenia with clinically assessed negative symptoms showed high levels of engagement, adherence, and acceptance over the 7-week intervention period with minimal risk to users. Moreover, results showed a within-group improvement in negative symptoms at the end of the study (Week 7) compared with baseline. Notably, participant engagement with the study app was not associated with negative symptom severity at baseline, suggesting that individuals would use CT-155 beta regardless of deficits in motivation and goal-directed behavior.

Participants demonstrated a high level of engagement, with 84% continuing to engage with CT-155 beta until the last day of the study. While engagement with DTx varies substantially across disease areas, our findings show that engagement with CT-155 beta was high in relation to other DTx studies, which typically range from 21% to 88%^54^. Furthermore, participant engagement and study completion rates reported in this study were higher than previous studies examining DTx for people with schizophrenia^46^. For example, 1 study examining smartphone delivery of CBT-based coaching for negative symptoms in people with schizophrenia initially reported acceptable DTx engagement and completion rates (e.g., above 80%), but then dropped by almost half after the first 4 weeks of the study^49^. Another DTx delivering CBT for negative symptoms found engagement rates as low as 19% by the end of the intervention period (24 weeks)^48^. Furthermore, no correlation was observed between multiple measures of engagement and the severity of ENS. This demonstrates that even at severe levels of ENS, where deficits in motivation and goal-directed behavior pose especially large barriers for accessing care, DTx, may be meaningful additions to treatment plans for those with schizophrenia. This high level of participant engagement with the study app reflects the end-of-study feedback in which participants found CT-155 beta engaging, visually appealing, and rich in high-quality content. Additionally, user experience surveys showed positive feedback, with 84% of participants stating they used CT-155 beta once or multiple times a day by Week 7. Furthermore, by the end of the 7-week intervention period, 95.5% of participants indicated they would recommend CT-155 beta to a friend. It is possible that aspects of the therapuetic played a role in driving engagement. Specifically, the CT-155 beta intervention was pointedly developed to establish and maintain a digital working alliance, akin to the therapeutic alliance in face-to-face treatment, which is linked with improved clinical outcomes in mental health^55–57^. The development of CT-155 beta was informed by an iterative patient-centered design process, which consisted of 2 phases during which feedback was captured from 4 peer support specialists with lived experiences of schizophrenia, and 15 patient panel participants with a diagnosis of schizophrenia^53^. Additionally, through an empathic style of communication, CT-155 beta provides core features of in-person therapeutic alliance to encourage daily adherence to lessons and activities by providing schizophrenia-specific psychoeducation and introducing core therapeutic skills. Like therapists getting to know their patient, CT-155 beta focuses on digital working alliance establishment through asking questions and rapport building, followed by a collaborative treatment approach and goal definition. These patient-centric features that support digital working alliance are subsequently integrated into all therapeutic components to optimize the experience by infusing an interactive, empathic, knowledgeable, and personally meaningful nature to the in-app experience that builds confidence for achieving treatment goals and is critical to treatment outcome. Findings from two single-arm, multicenter, exploratory studies show that a digital working alliance can be established and maintained between people with schizophrenia and CT-155 beta in independent cohorts^53^. Importantly, the formation of a positive digital working alliance was not limited by severe ENS, which supports the potential benefits of digital therapy for people experiencing severe symptoms. Furthermore, CT-155 beta was intended to be engaged with in small daily increments, as compared to the longer sessions (e.g., 20–50 min, once per week or less) that typically characterize face-to-face treatment. It may be that this type of daily engagement was particularly acceptable to this patient population. Furthermore, daily tasks were personalized based on participants’ feedback on how they were feeling on a particular day. For example, if a participant’s mood was low on a particular day, simpler tasks were suggested. Additionally, participants were permitted to skip a day during periods of low mood. The CT-155 beta app design may have contributed to engagement, as each goal was divided into four sequential steps, with a minimum of one day allocated for the completion of each step. As a result, regular engagement with the app was essential for progressing through the treatment. Finally, participants also received daily SMS from CT-155 beta as reminders to engage with the treatment during the intervention period. Overall, the high level of engagement observed in this study provides further evidence to suggest DTx can be feasible for individuals with mental illnesses, including schizophrenia^45,58–60^. However, our engagement data should be interpreted in the context of a clinical study environment where high engagement could be linked to the motivation of willing participants^61,62^. Further investigation of engagement with DTx in naturalistic conditions for the target population could be assessed through evidence collected through real-world studies.

In this study population, comparing ENS severity using CAINS-MAP from baseline to end of study (Week 7) across subjects showed notable improvement (17.6%). Previous research has reported that a CAINS-MAP total score of 17 or greater classifies negative symptoms as severe, persistent, and treatment resistant^63^, suggesting the improvement in negative symptoms to <17 in our study may represent a meaningful clinical improvement. A similar magnitude of improvement (3.5 point reduction in CAINS-MAP) was described in a previous study using mobile-assisted CBT intervention^48^. However, the within-subject change in both studies should be interpreted with caution, given the single-arm study design, but warrants further investigation. Furthermore, in the present study, the overall reduction in the CAINS-MAP total score was primarily driven by a change in 2 of the 3 sub-domains: social and recreational. There was no significant change observed in the work and school sub-domain, which is not surprising as changes in work and school situations are unlikely to happen in the short timeframe of the study. Additionally, the psychosocial interventions in CT-155 beta are designed to enhance goal-directed behavior, which is known to improve social and recreational skills^64,65^.

In this study, 24 out of 74 (32%) participants did not meet the strict eligibility criteria, particularly the moderate-to-severe ENS criterion (score of ≤30 on the MAP-SR), accounting for 12 of the 24 (50%) of screen failures. Screen fail rates vary across DTx studies in schizophrenia, with rates typically ranging from 14% to 66%^47–49,58,66,67^. It is important to note that study eligibility criteria mitigated potential confounding variables that may affect study assessments. For instance, individuals were excluded from participation if they were treated with more than 2 antipsychotic medications, had changed dose(s) of antipsychotic medication(s) within the last 3 months, were currently receiving inpatient treatment or psychotherapy, or had received inpatient treatment or psychotherapy within the last 3 months. Additionally, any beneficial effect of concomitant antipsychotics on ENS would presumably already have been observed at baseline. Therefore, any observed improvement in the severity of negative symptoms throughout the 7-week study is likely indicative of treatment benefit from CT‑155 beta. As clinical trials of DTx targeting ENS in people living with schizophrenia are uncommon, this study makes an important contribution by demonstrating improvements in ENS as a primary target rather than secondary improvements related to changes in positive symptoms.

It is important that the findings from this study should be interpreted in the context of its potential limitations. This was an exploratory single-arm study to test the feasibility of a beta version of the CT-155 app in a target population limited to 50 participants and not powered to detect improvement in ENS severity. Given the single-arm study design, within-subject change in ENS severity should be interpreted with caution and requires validation in a larger controlled study. The standard rigor of Phase 3 clinical study design includes but is not limited to (i) use of an appropriate control arm such as treatment-as-usual, waitlist control, or a digital control app^68^, (ii) appropriate blinding of the involved study and clinical site staff, and (iii) safety endpoints. These study design elements will allow DTx trials to be held to the same evidentiary standards as pharmacotherapy and strengthen study rigor. Importantly, in this exploratory single-arm study, a placebo effect due to spontaneous changes of the condition, expectancy beliefs of the patient, or the psychological and supportive benefits experienced during the intervention, cannot be excluded as possible reasons for observing potential effectiveness. The CONVOKE registrational study (NCT05838625) will provide a robust assessment to validate these preliminary effectiveness data. While the high patient engagement and adherence observed in this study is promising, it remains to be seen whether engagement (measured as time spent in the app or frequency of opening the app) and adherence (measured as proportion of therapeutic lessons and number of therapeutic goals completed) translate into clinical effectiveness in larger, statistically powered randomized controlled trials. Finally, the study size was limited, and findings may not generalize to a broader population of people living with schizophrenia. However, the demographics of study participants were generally consistent with those reported in schizophrenia population studies, including a higher incidence in males^69,70^, a predominance of participants identifying as Black or African American—reflecting the higher reported diagnostic rates in this group^7^, and a majority having an education level of high school or below^71^. In addition, almost all (94%) participants in this study reported an annual income under US$25,000, which likely reflects low employment rates in people with schizophrenia^72^. Furthermore, the inclusion criteria for participants to own their own mobile device may have biased the sample toward a more digitally proficient population, and may therefore not be reflective of patients who do not own a mobile device and who potentially may have a lower device proficiency. However, this inclusion criterion was critical to ensure adequate fit between participant abilities (e.g., digital literacy) and engagement demands of CT-155 beta. Doing so ensured that findings were due to the intervention rather than user error, thereby strengthening the internal validity of the findings.

Despite these limitations, this study demonstrated the feasibility of CT-155 beta, highlighting the ability of patients with schizophrenia to engage with the digital intervention and an associated reduction in ENS severity from baseline. Findings from this exploratory study show the potential of CT-155 beta to be a viable adjunct to current treatment modalities in an area with a pressing need for new and effective therapies. The improvement in ENS observed after 7 weeks of CT-155 beta suggests that digital treatments may help alleviate the significant disease burden associated with ENS in schizophrenia. Although these findings are encouraging, they are exploratory, and a large Phase III study will validate these initial insights (CONVOKE). Nevertheless, the results contribute to a growing body of evidence that shows that people living with schizophrenia are not only capable and willing to use technology to support their treatment but also benefit from these opportunities. Given the urgent need for innovative, effective, and accessible treatments to enhance standard care and mental health services for schizophrenia, particularly for negative symptoms, evidence-based prescription DTx could provide a promising solution. By leveraging the widespread use of smartphones, DTx has the potential to deliver high-quality, standardized, and scalable interventions to individuals with schizophrenia.

## Methods

### Study design

**Fig. 5:**
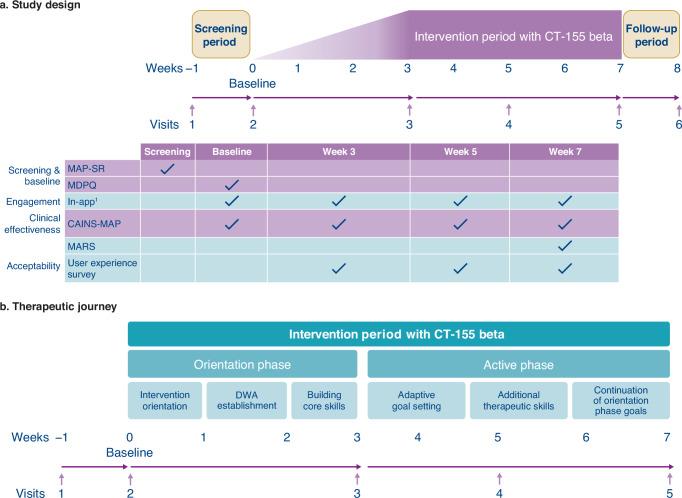
Study design and therapeutic journey. **a** Overview of study design (CT-155-C-002; NCT05486312) and **b** therapeutic journey of participants engaging with CT-155 beta. ^1^Participant engagement and adherence with the study app (CT‑155 beta) were measured passively through the app. Engagement was defined as the number of app opens and sessions of 60 s or greater. Adherence was defined as the number of lessons and goals completed. An independent single-arm, multicenter, exploratory study (CT-155-C-002/NCT05486312) was conducted across ten sites in the United States. The study comprised a screening period, an intervention period, and a follow-up period. The intervention period comprised a 3-week orientation phase with an additional 4-week active phase. The orientation phase included experiences to provide intervention orientation, digital working alliance establishment, and build core therapeutic skills necessary for later stages of intervention. Goals were comprised of 4 steps, where each step is allotted a day to complete. Therefore, 1 goal required a minimum of 4 days to complete. Goal-centered tasks were defined as completing an in-app activity or reporting the completion of the activity during the Activity check-in. CAINS-MAP clinical assessment interview for negative symptoms motivation and pleasure scale, CT-155 beta a beta version of CT-155/BI 3972080, DWA digital working alliance, ENS experiential negative symptoms, MAP-SR motivation and pleasure scale—self report, MARS mobile app rating scale, MDPQ mobile device proficiency questionnaire.

This multicenter, single-arm, exploratory study (clinicaltrial.gov: NCT05486312) was conducted across 9 sites in the United States between July 2022 and November 2022. The study was approved by the Western Copernicus Group IRB (approval number: 20220609), with written informed consent obtained prior to participation. During an in-person screening visit (Visit 1), site personnel assisted with the download and set-up of CT-155 beta onto participants’ personal smartphone devices. CT-155 beta was activated following confirmation of eligibility during baseline visit (Fig. 5A). During the 7-week intervention period, participants attended in-person clinic visits at Week 3 (Visit 3), Week 5 (Visit 4), and Week 7 (Visit 5). Further details on data privacy can be found in the Supplementary Materials. Participants were offered up to US$735 for partaking in the study. Incentives were offered for attending study visits, but not for daily app usage.

### Participants

Eligible participants were ≥18 years of age with a primary diagnosis of schizophrenia for at least 1 year (as per the Diagnostic and Statistical Manual of Mental Disorders, fifth edition [DSM-5]), and moderate-to-severe ENS (defined at screening as a score of ≤30 on the MAP-SR^73^). The MAP-SR is a 15-item self-report scale where participants are asked to rate their deficits in motivation and pleasure across social, recreational, or work domains. Items are rated on a 5-point Likert scale with lower scores indicating greater pathology. Additionally, participants must have been receiving a stable dose of a maximum of two different antipsychotic medications for ≥12 weeks prior to screening. Exclusion criteria included a DSM‑5 or International Classification of Diseases 10th edition diagnosis other than schizophrenia, and/or a substance or alcohol use disorder (excluding caffeine and nicotine). Participants receiving clozapine, haloperidol, or who had received psychotherapy within 12 weeks of screening were excluded. Finally, participants with active prominent positive symptoms that, in the opinion of the investigator, would preclude effective engagement in treatment for negative symptoms were also excluded. The complete list of eligibility criteria is included in the Supplementary Materials. A list of assessments conducted at screening and baseline are described in Supplementary Table 4.

### Intervention

CT-155 is currently considered an investigational medical device per FDA regulations, and more specifically, a software-as-a-medical device to treat ENS in people living with schizophrenia^50,74^. Individual components of CT-155 were designed to incorporate principles of evidence-based, in-person psychosocial therapy to target ENS^75–78^. CT-155 beta was an abbreviated 7-week version of a full intervention experience with CT‑155. The design and development of CT-155 beta were informed by participant feedback gathered during interviews with people living with schizophrenia. Further details on the participant-centered design process have been described further in Snipes et al, *JMIR Mental Health* 2025^53^.

The overall intervention period (7 weeks) comprised a 3-week orientation phase, a 4-week active phase, and concluded with a 1-week follow-up period during which a remote teleconference occurred (Fig. 5A). The orientation phase of the intervention included lessons and activities designed to establish a therapeutic alliance between participants and CT-155 beta (i.e., a digital working alliance^53^) and build core therapeutic skills (including distress tolerance) necessary for later stages of the intervention (Fig. 5B). Participants had access to a total of 21 therapeutic lessons (18 required core lessons and 3 optional lessons) in the orientation phase. The active phase included continuation of the orientation phase goals, with incorporated adaptive goal-setting activities, and additional therapeutic skills training (Fig. 5B). Participants were encouraged to complete lessons and activities in CT-155 beta daily and were asked to complete a daily check‑in. In this phase, therapeutic techniques are delivered daily to help participants set goals (adaptive goal setting) that encourage real-world engagement (behavioral activation) while removing barriers (cognitive restructuring) and providing skills to facilitate goal attainment (positive affect training). The adaptive goal-setting modules focused on delivering interventions known to target negative symptoms in important functional areas (i.e., social items, work and school items, recreation items). As part of adaptive goal setting, activities containing behavioral activation elements were adaptively set and gradually increased in difficulty as participants acquired skills. Participants received normalizing messages and cognitive or behavioral strategies to help manage negative experiences related to cognition, beliefs, emotions, and associated behaviors. Furthermore, the positive affect training component of CT-155 beta taught participants skills derived from positive psychology and affective science to target ENS by increasing positive affect.

### Study assessments

#### Participant engagement and adherence with CT-155 beta

Participant engagement with CT‑155 beta was measured passively by the smartphone as a result of participants' day-to-day app usage and completion of in-app activities. These included the number of days that CT-155 beta was opened (any usage event recorded during a given day), the number of daily check-ins completed, and the duration of each CT-155 beta use session. Study retention was assessed using Kaplan–Meier retention analysis of last app open data until the end of the study period. Adherence was determined by assessing the number of available therapeutic lessons completed during the orientation phase and the number of goal-centered tasks completed and goals successfully accomplished during the active phase of the study.

#### Negative symptoms

The potential of CT-155 beta to reduce the ENS of schizophrenia was assessed by the change in the CAINS-MAP total score from baseline to Week 7. CAINS is a second-generation negative symptom scale^15^ that has demonstrated reliability, convergent and discriminant validity, and has a short test-retest administration time of 15–30 min^15,79,80^. CAINS was originally developed on the recommendations of an expert group convened by the National Institute of Mental Health and the Measurement and Treatment Research to Improve Cognition in Schizophrenia initiative to reconceptualize negative symptoms to include 5 domains: avolition, asociality, alogia, anhedonia, and blunted affect^81^. The CAINS consists of 2 subscales covering the 5 domains: Motivation and Pleasure (MAP; i.e., avolition, anhedonia, and asociality), and Expressivity (EXP; i.e., blunted affect, alogia) (Supplementary Table 5)^15^. CAINS offers a more detailed assessment of negative symptoms of schizophrenia compared with older scales such as the Positive and Negative Syndrome Scale (PANSS), which treats these symptoms as a more generalized construct^16^. It focuses on experiential deficits by using detailed probes to assess internal experiences such as joy, pleasure, interest, or intimacy, instead of simply relying on behavior. CAINS underwent multiple development and psychometric testing phases, where it has been validated against patient-reported experiences, in-the-moment experiences, and behavioral observations. CAINS-MAP total scores were grouped according to baseline ENS symptom severity. In the present study, CAINS-MAP was administered in-clinic by trained raters. In line with FDA's current best practices and terminology in medical device clinical research, improvement in clinical outcomes is herein referred to as effectiveness (rather than efficacy)^82^.

#### Acceptability and user experience of CT-155 beta

Acceptability and user experience of CT-155 beta were assessed using the validated MARS^83^ and a brief non-validated user experience survey, respectively. At the end of the study (Week 7), participants rated the quality of CT-155 beta through MARS (Supplementary Table 6) and shared their expectation of the therapeutic benefit of CT-155 beta via a brief 3-question survey. MARS items were rated from 1 (inadequate) to 5 (excellent), and a mean score per domain calculated^83^. In the user experience survey, participants were asked at Weeks 3, 5, and 7 how often they used CT-155 beta and whether they would recommend CT-155 beta to a friend. Three free-response questions covering the benefits of, frustrations with, and suggested improvements to CT-155 beta were also asked. A modified inductive approach was used to assess free-text responses with themes emerging directly from the data without predetermined categories.

#### Safety

Safety endpoints included the frequency and severity of all AEs, serious AEs, and discontinuations from the study due to AEs. These were collected from when informed consent was provided until the end of the follow-up period.

### Statistical analysis

This was an exploratory study to assess participant engagement and adherence with CT-155 beta, along with potential effectiveness and user feedback to determine the acceptability of CT-155 beta. As hypothesis testing was not the objective of this study, no formal a priori power calculations were performed. All analyses were performed on the observed data, and no imputations were performed for missing data. Descriptive statistics were evaluated for demographic data and study variables in all participants who enrolled in the study. To examine overall retention in the study, we used the univariate Kaplan–Meier survival curve^84^. Within-subject changes in CAINS-MAP scores at baseline were compared with those at Week 7 using a Student’s paired *t*-test. For correlation analyses, a Spearman rank-order correlation coefficient was calculated together with the 95% two-sided CIs.

## Supplementary information

Supplemental material

## Data Availability

To ensure independent interpretation of clinical study results and enable authors to fulfill their role and obligations under the ICMJE criteria, Boehringer Ingelheim grants all external authors access to relevant clinical study data. In adherence with the Boehringer Ingelheim Policy on Transparency and Publication of Clinical Study Data, scientific and medical researchers can request access to clinical study data, typically, 1 year after the approval has been granted by major Regulatory Authorities or after termination of the development program. Researchers should use the https://vivli.org/ link to request access to study data and visit https://www.mystudywindow.com/msw/datasharing for further information.

## References

[1] World Health Organization. *Schizophrenia*, https://www.who.int/news-room/fact-sheets/detail/schizophrenia (2022).

[2] Kessler, R. C. et al. The prevalence and correlates of nonaffective psychosis in the National Comorbidity Survey Replication (NCS-R). *Biol. Psychiatry* **58**, 668–676 (2005). 16023620 10.1016/j.biopsych.2005.04.034 PMC2847859

[3] Wu, E. Q., Shi, L., Birnbaum, H., Hudson, T. & Kessler, R. Annual prevalence of diagnosed schizophrenia in the USA: a claims data analysis approach. *Psychol. Med.* **36**, 1535–1540 (2006). 16907994 10.1017/S0033291706008191

[4] Desai, P. R., Lawson, K. A., Barner, J. C. & Rascati, K. L. Estimating the direct and indirect costs for community-dwelling patients with schizophrenia. *J. Pharm. Health Serv. Res.* **4**, 187–194 (2013).

[5] Charlson, F. J. et al. Global epidemiology and burden of schizophrenia: findings from the global burden of disease Study 2016. *Schizophr. Bull.* **44**, 1195–1203 (2018). 29762765 10.1093/schbul/sby058 PMC6192504

[6] Kotzeva, A., Mittal, D., Desai, S., Judge, D. & Samanta, K. Socioeconomic burden of schizophrenia: a targeted literature review of types of costs and associated drivers across 10 countries. *J. Med. Econ.* **26**, 70–83 (2023). 36503357 10.1080/13696998.2022.2157596

[7] Olbert, C. M., Nagendra, A. & Buck, B. Meta-analysis of Black vs. White racial disparity in schizophrenia diagnosis in the United States: Do structured assessments attenuate racial disparities?. *J. Abnorm. Psychol.* **127**, 104–115 (2018). 29094963 10.1037/abn0000309

[8] Schwartz, R. C. & Blankenship, D. M. Racial disparities in psychotic disorder diagnosis: a review of empirical literature. *World J. Psychiatry* **4**, 133–140 (2014). 25540728 10.5498/wjp.v4.i4.133 PMC4274585

[9] Bobes, J., Arango, C., Garcia-Garcia, M. & Rejas, J. Prevalence of negative symptoms in outpatients with schizophrenia spectrum disorders treated with antipsychotics in routine clinical practice: findings from the CLAMORS study. *J. Clin. Psychiatry* **71**, 280–286 (2010). 19895779 10.4088/JCP.08m04250yel

[10] Sicras-Mainar, A., Maurino, J., Ruiz-Beato, E. & Navarro-Artieda, R. Impact of negative symptoms on healthcare resource utilization and associated costs in adult outpatients with schizophrenia: a population-based study. *BMC Psychiatry* **14**, 225 (2014). 25096022 10.1186/s12888-014-0225-8 PMC4149268

[11] Fusar-Poli, P. et al. Treatments of negative symptoms in schizophrenia: meta-analysis of 168 randomized placebo-controlled trials. *Schizophr. Bull.* **41**, 892–899 (2015). 25528757 10.1093/schbul/sbu170 PMC4466178

[12] Galderisi, S. et al. EPA guidance on treatment of negative symptoms in schizophrenia. *Eur. Psychiatry* **64**, e21 (2021). 33726883 10.1192/j.eurpsy.2021.13 PMC8057437

[13] Correll, C. U. et al. Systematic literature review of schizophrenia clinical practice guidelines on acute and maintenance management with antipsychotics. *Schizophrenia* **8**, 5 (2022). 35210430 10.1038/s41537-021-00192-x PMC8873492

[14] Govil, P. & Kantrowitz, J. T. Negative symptoms in schizophrenia: an update on research assessment and the current and upcoming treatment landscape. *CNS Drugs* **39**, 243–262(2025). 10.1007/s40263-024-01151-7 39799532

[15] Kring, A. M., Gur, R. E., Blanchard, J. J., Horan, W. P. & Reise, S. P. The clinical assessment interview for negative symptoms (CAINS): final development and validation. *Am. J. Psychiatry* **170**, 165–172 (2013). 23377637 10.1176/appi.ajp.2012.12010109 PMC3785242

[16] Kirkpatrick, B., Fenton, W. S., Carpenter, W. T. Jr. & Marder, S. R. The NIMH-MATRICS consensus statement on negative symptoms. *Schizophr. Bull.* **32**, 214–219 (2006). 16481659 10.1093/schbul/sbj053 PMC2632223

[17] Correll, C. U. & Schooler, N. R. Negative symptoms in schizophrenia: a review and clinical guide for recognition, assessment, and treatment. *Neuropsychiatr. Dis. Treat.* **16**, 519–534 (2020). 32110026 10.2147/NDT.S225643 PMC7041437

[18] Moses, D. G., Palaniappan, P. & Ponraj, P. C. Residual experiential symptoms mediate the effect of expressive symptoms over the social functioning in remitted schizophrenia. *Ind. Psychiatry J.* **32**, 309–316 (2023). 38161473 10.4103/ipj.ipj_30_23 PMC10756619

[19] Liemburg, E. J. et al. Expressive deficits and amotivation as mediators of the associations between cognitive problems and functional outcomes: Results from two independent cohorts. *Schizophr. Res.* **218**, 283–291 (2020). 31948899 10.1016/j.schres.2019.12.018

[20] Strauss, G. P. et al. Deconstructing negative symptoms of schizophrenia: avolition–apathy and diminished expression clusters predict clinical presentation and functional outcome. *J. Psychiatr. Res.* **47**, 783–790 (2013). 23453820 10.1016/j.jpsychires.2013.01.015 PMC3686506

[21] Llerena, K., Reddy, L. F. & Kern, R. S. The role of experiential and expressive negative symptoms on job obtainment and work outcome in individuals with schizophrenia. *Schizophr. Res.* **192**, 148–153 (2018). 28599750 10.1016/j.schres.2017.06.001

[22] Savill, M. et al. The relationship between experiential deficits of negative symptoms and subjective quality of life in schizophrenia. *Schizophr. Res.* **176**, 387–391 (2016). 27328889 10.1016/j.schres.2016.06.017

[23] Gold, J. M. et al. Negative symptoms and the failure to represent the expected reward value of actions: behavioral and computational modeling evidence. *Arch. Gen. Psychiatry* **69**, 129–138 (2012). 22310503 10.1001/archgenpsychiatry.2011.1269 PMC4406055

[24] Filip, T. F. et al. Defeatist performance beliefs in individuals with recent-onset schizophrenia: Relationships with cognition and negative symptoms. *Schizophr. Res.* **270**, 212–219 (2024). 38924939 10.1016/j.schres.2024.06.021 PMC11323074

[25] Grant, P. M., Huh, G. A., Perivoliotis, D., Stolar, N. M. & Beck, A. T. Randomized trial to evaluate the efficacy of cognitive therapy for low-functioning patients with schizophrenia. *Arch. Gen. Psychiatry* **69**, 121–127 (2012). 21969420 10.1001/archgenpsychiatry.2011.129

[26] Granholm, E., Holden, J. & Worley, M. Improvement in negative symptoms and functioning in cognitive-behavioral social skills training for schizophrenia: mediation by defeatist performance attitudes and asocial beliefs. *Schizophr. Bull.* **44**, 653–661 (2018). 29036391 10.1093/schbul/sbx099 PMC5890456

[27] McDonagh, M. S. et al. Psychosocial Interventions for adults with schizophrenia: an overview and update of systematic reviews. *Psychiatr. Serv.* **73**, 299–312 (2022). 34384230 10.1176/appi.ps.202000649

[28] Keepers, G. A. et al. The American Psychiatric Association practice guideline for the treatment of patients with schizophrenia. *Focus* **18**, 493–497 (2020). 33343262 10.1176/appi.focus.18402 PMC7725162

[29] National Collaborating Centre for Mental Health. In *Psychosis and Schizophrenia in Adults: Treatment and Management: Updated Edition 2014* (National Institute for Health and Care Excellence (UK). Copyright ©, National Collaborating Centre for Mental Health, 2014). 25340235

[30] Barlati, S., Nibbio, G. & Vita, A. Evidence-based psychosocial interventions in schizophrenia: a critical review. *Curr. Opin. Psychiatry* **37**, 131–139 (2024). 38410981 10.1097/YCO.0000000000000925 PMC10990032

[31] Granholm, E. & Harvey, P. D. Social skills training for negative symptoms of schizophrenia. *Schizophr. Bull.* **44**, 472–474 (2018). 29315427 10.1093/schbul/sbx184 PMC5890477

[32] Cella, M. et al. Psychosocial and behavioural interventions for the negative symptoms of schizophrenia: a systematic review of efficacy meta-analyses. *Br. J. Psychiatry* **223**, 321–331 (2023). 36919340 10.1192/bjp.2023.21 PMC10331321

[33] Haddock, G. et al. An investigation of the implementation of NICE-recommended CBT interventions for people with schizophrenia. *J. Ment. Health* **23**, 162–165 (2014). 24433132 10.3109/09638237.2013.869571

[34] Kopelovich, S. L., Strachan, E., Sivec, H. & Kreider, V. Stepped care as an implementation and service delivery model for cognitive behavioral therapy for psychosis. *Community Ment. Health J.* **55**, 755–767 (2019). 30623294 10.1007/s10597-018-00365-6

[35] Macbeth, A., Gumley, A., Schwannauer, M. & Fisher, R. Service engagement in first episode psychosis: clinical and premorbid correlates. *J. Nerv. Ment. Dis.* **201**, 359–364 (2013). 23588222 10.1097/NMD.0b013e31828e0e19

[36] International Organization for Standardization (ISO). *ISO/TR 11147:2023(en) Health informatics — Personalized Digital Health — Digital Therapeutics Health Software Systems* https://www.iso.org/obp/ui/en/#iso:std:iso:tr:11147:ed-1:v1:en (2023).

[37] Chung, J. Y. Digital therapeutics and clinical pharmacology. *Transl. Clin. Pharmacol.* **27**, 6–11 (2019). 32055575 10.12793/tcp.2019.27.1.6 PMC6989269

[38] Achtyes, E. D. et al. Off-hours use of a smartphone intervention to extend support for individuals with schizophrenia spectrum disorders recently discharged from a psychiatric hospital. *Schizophr. Res.* **206**, 200–208 (2019). 30551981 10.1016/j.schres.2018.11.026

[39] Cuyler, R. N., Katdare, R., Thomas, S. & Telch, M. J. Real-world outcomes of an innovative digital therapeutic for treatment of panic disorder and PTSD: A 1,500 patient effectiveness study. *Front. Digit. Health* **4**, 976001 (2022). 36465089 10.3389/fdgth.2022.976001 PMC9712796

[40] Davenport, N. D. & Werner, J. K. A randomized sham-controlled clinical trial of a novel wearable intervention for trauma-related nightmares in military veterans. *J. Clin. Sleep Med.* **19**, 361–369 (2023). 36305584 10.5664/jcsm.10338 PMC9892731

[41] Bickel, W. K., Marsch, L. A., Buchhalter, A. R. & Badger, G. J. Computerized behavior therapy for opioid-dependent outpatients: a randomized controlled trial. *Exp. Clin. Psychopharmacol.* **16**, 132–143 (2008). 18489017 10.1037/1064-1297.16.2.132 PMC2746734

[42] Maricich, Y. A., Nunes, E. V., Campbell, A. N. C., Botbyl, J. D. & Luderer, H. F. Safety and efficacy of a digital therapeutic for substance use disorder: secondary analysis of data from a NIDA clinical trials network study. *Subst. Abus.* **43**, 937–942 (2022). 35420979 10.1080/08897077.2022.2060425

[43] Kollins, S. H., Childress, A., Heusser, A. C. & Lutz, J. Effectiveness of a digital therapeutic as adjunct to treatment with medication in pediatric ADHD. *NPJ Digit. Med.* **4**, 58 (2021). 33772095 10.1038/s41746-021-00429-0 PMC7997870

[44] Kirk, H. E., Spencer-Smith, M., Wiley, J. F. & Cornish, K. M. Gamified attention training in the primary school classroom: a cluster-randomized controlled trial. *J. Atten. Disord.* **25**, 1146–1159 (2021). 31718386 10.1177/1087054719887435

[45] Ben-Zeev, D. et al. Feasibility, acceptability, and preliminary efficacy of a smartphone intervention for schizophrenia. *Schizophr. Bull.* **40**, 1244–1253 (2014). 24609454 10.1093/schbul/sbu033 PMC4193714

[46] Bucci, S. et al. Actissist: proof-of-concept trial of a theory-driven digital intervention for psychosis. *Schizophr. Bull.* **44**, 1070–1080 (2018). 29566206 10.1093/schbul/sby032 PMC6135229

[47] Fulford, D. et al. Preliminary outcomes of an ecological momentary intervention for social functioning in schizophrenia: pre-post study of the motivation and skills support app. *JMIR Ment. Health* **8**, e27475 (2021). 34128812 10.2196/27475 PMC8277369

[48] Granholm, E. et al. Mobile-assisted cognitive behavioral therapy for negative symptoms: open single-arm trial with schizophrenia patients. *JMIR Ment. Health* **7**, e24406 (2020). 33258792 10.2196/24406 PMC7738249

[49] Schlosser, D. A. et al. Efficacy of PRIME, a mobile app intervention designed to improve motivation in young people with schizophrenia. *Schizophr. Bull.* **44**, 1010–1020 (2018). 29939367 10.1093/schbul/sby078 PMC6101497

[50] Boehringer Ingelheim. *Boehringer Ingelheim and Click Therapeutics Receive FDA Breakthrough Device Designation for Schizophrenia Prescription Digital Therapeutic*, https://www.boehringer-ingelheim.com/human-health/mental-disorders/schizophrenia/fda-grants-breakthrough-status-schizophrenia-2 (2024).

[51] US Food and Drug Administration. Breakthrough devices program*. Guidance for Industry and Food and Drug Administration Staff* https://www.fda.gov/media/162413/download (2023).

[52] US Food and Drug Administration. *Patient-Focused Drug Development: Collecting Comprehensive and Representative Input* https://www.fda.gov/regulatory-information/search-fda-guidance-documents/patient-focused-drug-development-collecting-comprehensive-and-representative-input (2018).

[53] Snipes, C. et al. Establishment and maintenance of a digital therapeutic alliance in people living with negative symptoms of schizophrenia: two exploratory single-arm studies. *JMIR Ment. Health* **12**, e64959 (2025). 39869902 10.2196/64959 PMC11811661

[54] Boucher, E. M. & Raiker, J. S. Engagement and retention in digital mental health interventions: a narrative review. *BMC Digit. Health* **2**, 52 (2024).

[55] Flückiger, C., Del Re, A. C., Wampold, B. E., Symonds, D. & Horvath, A. O. How central is the alliance in psychotherapy? A multilevel longitudinal meta-analysis. *J. Couns. Psychol.* **59**, 10–17 (2012). 21988681 10.1037/a0025749

[56] Flückiger, C., Del Re, A. C., Wampold, B. E. & Horvath, A. O. The alliance in adult psychotherapy: a meta-analytic synthesis. *Psychotherapy ((Chic))* **55**, 316–340 (2018). 29792475 10.1037/pst0000172

[57] Ardito, R. B. & Rabellino, D. Therapeutic alliance and outcome of psychotherapy: historical excursus, measurements, and prospects for research. *Front. Psychol.* **2**, 270 (2011). 22028698 10.3389/fpsyg.2011.00270 PMC3198542

[58] Bell, I. H. et al. Pilot randomised controlled trial of a brief coping-focused intervention for hearing voices blended with smartphone-based ecological momentary assessment and intervention (SAVVy): feasibility, acceptability and preliminary clinical outcomes. *Schizophr. Res.* **216**, 479–487 (2020). 31812327 10.1016/j.schres.2019.10.026

[59] Ben-Zeev, D. et al. mHealth for schizophrenia: patient engagement with a mobile phone intervention following hospital discharge. *JMIR Ment. Health* **3**, e34 (2016). 27465803 10.2196/mental.6348 PMC4999306

[60] Dabit, S., Quraishi, S., Jordan, J. & Biagianti, B. Improving social functioning in people with schizophrenia-spectrum disorders via mobile experimental interventions: results from the CLIMB pilot trial. *Schizophr. Res. Cogn.* **26**, 100211 (2021). 34381699 10.1016/j.scog.2021.100211 PMC8340304

[61] Baumel, A., Edan, S. & Kane, J. M. Is there a trial bias impacting user engagement with unguided e-mental health interventions? A systematic comparison of published reports and real-world usage of the same programs. *Transl. Behav. Med.* **9**, 1020–1033 (2019). 31689344 10.1093/tbm/ibz147

[62] Lipschitz, J. M., Pike, C. K., Hogan, T. P., Murphy, S. A. & Burdick, K. E. The engagement problem: a review of engagement with digital mental health interventions and recommendations for a path forward. *Curr. Treat. Options Psychiatry* **10**, 119–135 (2023). 38390026 10.1007/s40501-023-00297-3 PMC10883589

[63] Li, Y. et al. Revisiting the persistent negative symptoms proxy score using the clinical assessment interview for negative symptoms. *Schizophr. Res.* **202**, 248–253 (2018). 29996973 10.1016/j.schres.2018.07.005

[64] Jacob, J., Stankovic, M., Spuerck, I. & Shokraneh, F. Goal setting with young people for anxiety and depression: What works for whom in therapeutic relationships? A literature review and insight analysis. *BMC Psychol.* **10**, 171 (2022). 35831897 10.1186/s40359-022-00879-5 PMC9281142

[65] Tryon, G. S., Birch, S. E. & Verkuilen, J. Meta-analyses of the relation of goal consensus and collaboration to psychotherapy outcome. *Psychotherapy* **55**, 372–383 (2018). 30335451 10.1037/pst0000170

[66] Ghaemi, S. N., Sverdlov, O., van Dam, J., Campellone, T. & Gerwien, R. A smartphone-based intervention as an adjunct to standard-of-care treatment for schizophrenia: randomized controlled trial. *JMIR Form. Res.* **6**, e29154 (2022). 35343910 10.2196/29154 PMC9002609

[67] Garety, P. et al. Effects of SlowMo, a blended digital therapy targeting reasoning, on paranoia among people with psychosis: a randomized clinical trial. *JAMA Psychiatry* **78**, 714–725 (2021). 33825827 10.1001/jamapsychiatry.2021.0326 PMC8027943

[68] Lutz, J., Offidani, E., Taraboanta, L., Lakhan, S. E. & Campellone, T. R. Appropriate controls for digital therapeutic clinical trials: a narrative review of control conditions in clinical trials of digital therapeutics (DTx) deploying psychosocial, cognitive, or behavioral content. *Front. Digit. Health* **4**, 823977 (2022). 36060538 10.3389/fdgth.2022.823977 PMC9436387

[69] He, H. et al. Trends in the incidence and DALYs of schizophrenia at the global, regional and national levels: results from the Global Burden of Disease Study 2017. *Epidemiol. Psychiatr. Sci.* **29**, e91 (2020). 31928566 10.1017/S2045796019000891 PMC7214712

[70] Li, X., Zhou, W. & Yi, Z. A glimpse of gender differences in schizophrenia. *Gen. Psychiatr.* **35**, e100823 (2022). 36118418 10.1136/gpsych-2022-100823 PMC9438004

[71] Holm, M., Taipale, H., Tanskanen, A., Tiihonen, J. & Mitterdorfer-Rutz, E. Employment among people with schizophrenia or bipolar disorder: a population-based study using nationwide registers. *Acta Psychiatr. Scand.* **143**, 61–71 (2021). 33155273 10.1111/acps.13254 PMC7839734

[72] Kadakia, A. et al. The economic burden of schizophrenia in the United States. *J. Clin. Psychiatry* **83**, 22m14458 (2022). 10.4088/JCP.22m14458 36244006

[73] Llerena, K. et al. The motivation and pleasure scale-self-report (MAP-SR): reliability and validity of a self-report measure of negative symptoms. *Compr. Psychiatry* **54**, 568–574 (2013). 23351831 10.1016/j.comppsych.2012.12.001 PMC4762003

[74] International Medical Device Regulators Forum. *Software as a Medical Device (SaMD): Key Definitions* https://www.imdrf.org/sites/default/files/docs/imdrf/final/technical/imdrf-tech-131209-samd-key-definitions-140901.pdf (2013).

[75] Avasthi, A., Sahoo, S. & Grover, S. Clinical practice guidelines for cognitive behavioral therapy for psychotic disorders. *Indian J. Psychiatry* **62**, S251–s262 (2020). 32055067 10.4103/psychiatry.IndianJPsychiatry_774_19 PMC7001360

[76] Sivec, H. J. & Montesano, V. L. Cognitive behavioral therapy for psychosis in clinical practice. *Psychotherapy* **49**, 258–270 (2012). 22642528 10.1037/a0028256

[77] Morrison, A. K. Cognitive behavior therapy for people with schizophrenia. *Psychiatry* **6**, 32–39 (2009). 20104290 PMC2811142

[78] Tai, S. & Turkington, D. The evolution of cognitive behavior therapy for schizophrenia: current practice and recent developments. *Schizophr. Bull.* **35**, 865–873 (2009). 19661198 10.1093/schbul/sbp080 PMC2728828

[79] Blanchard, J. J. et al. Examining the reliability and validity of the clinical assessment interview for negative symptoms within the management of schizophrenia in clinical practice (MOSAIC) multisite national study. *Schizophr. Res.* **185**, 137–143 (2017). 28087270 10.1016/j.schres.2017.01.011

[80] Blanchard, J. J. et al. Motivation and pleasure deficits undermine the benefits of social affiliation in psychosis. *Clin. Psychol. Sci.* **12**, 1195–1217 (2024). 39635455 10.1177/21677026241227886 PMC11617013

[81] Marder, S. R. & Fenton, W. Measurement and treatment research to improve cognition in schizophrenia: NIMH MATRICS initiative to support the development of agents for improving cognition in schizophrenia. *Schizophr. Res.* **72**, 5–9 (2004). 15531402 10.1016/j.schres.2004.09.010

[82] US Food and Drug Administration. *CFR*—*Code of Federal Regulations Title**21* https://www.accessdata.fda.gov/scripts/cdrh/cfdocs/cfcfr/CFRSearch.cfm?FR=860.7 (2024).

[83] Stoyanov, S. R. et al. Mobile app rating scale: a new tool for assessing the quality of health mobile apps. *JMIR Mhealth Uhealth* **3**, e27 (2015). 25760773 10.2196/mhealth.3422 PMC4376132

[84] Rich, J. T. et al. A practical guide to understanding Kaplan–Meier curves. *Otolaryngol. Head Neck Surg.* **143**, 331–336 (2010). 20723767 10.1016/j.otohns.2010.05.007 PMC3932959

