# Supplementary material for: Feasibility of a digital therapeutic for experiential negative symptoms of schizophrenia: results from an exploratory study
Source: Schizophrenia (Heidelb). 2025 Sep 26;11(1):120. doi: 10.1038/s41537-025-00659-1 (PMC12475125; doi:10.1038/s41537-025-00659-1)
Supplement: Supplementary file 1 — Supplemental material [file 41537_2025_659_MOESM1_ESM.pdf]

Supplementary materials

Participant data privacy

To ensure participants' personal data and privacy were protected throughout the study, the study was conducted in compliance with applicable laws and accepted security standards, including the Health Insurance Portability and Accountability Act of 1996, the U.S. National Institute of Standards and Technology Framework, and the Service Organization Control Type 2 controls. This ensured the security, confidentiality, integrity, and availability of the assets and other sensitive information that was collected, used, and maintained from participants. It also protected against any anticipated threats or hazards to the security, integrity, or availability of such information and maintained information security controls that were appropriate to the size, scope, and business of Click Therapeutics, who designed CT-155 beta. These measures also maintained safeguards and controls to protect information from loss, theft, destruction, unauthorized manipulation, disclosure, or unavailability. Industry best practices for data protection and risk management were followed, and internal controls, governance and oversight, as well as layered security controls were implemented throughout. Data were classified, managed, processed, and stored in accordance with the local data protection law and other regulations.

Participants were assigned a unique identifier to protect their identity and were informed that their personal study-related data will be used by the sponsor in accordance with local data protection law and that their medical records may be examined by Clinical Quality Assurance auditors or other authorized personnel appointed by the sponsor, by appropriate Institutional Review Board/Independent Ethics Committee members, and/or by inspectors from regulatory authorities.

The compliance program was designed to: 1) ensure the security, confidentiality, integrity, and availability of the assets and other sensitive information that was collected, used, and maintained; 2) protect against any anticipated threats or hazards to the security, integrity, or availability of such information; 3) maintain information security controls; 4) maintain safeguards and controls to protect information from loss, theft, destruction, unauthorized manipulation, disclosure, or unavailability.

Inclusion Criteria

A participant was eligible for entry into the study if all the following inclusion criteria were met:

- Was willing and able to provide written informed consent to participate in the study, attend study visits, and comply with study-related requirements and assessments
- Was ≥18 of age at the time of informed consent
- Was fluent in written and spoken English, confirmed by ability to read and understand the informed consent form
- Had a primary diagnosis of schizophrenia using the diagnostic criteria for schizophrenia as defined in the Diagnostic and Statistical Manual of Mental Disorders, fifth edition (DSM-5), for at least 1-year prior to screening
- Was in the stable phase of illness, as assessed by the investigator after review of medical records or documented discussion with the treating physician
- Had outpatient treatment status at the time of screening, with no inpatient treatment for schizophrenia within 12 weeks prior to screening
- Was on a stable dose of antipsychotic medication(s) for at least 12 weeks prior to enrollment (Day 1), as determined by the investigator
- Had obtained a score of 30 or less on the Motivation and Pleasure – Self Report, as assessed at the screening visit
- Was the sole user, per participant self-report, of an iPhone with an iPhone operating system (iOS) 13 or greater, or a smartphone with an Android operating system 10 or greater, and was willing to download and use the beta version of CT-155/BI 3972080 (CT-155 beta) as required per the protocol
- Was the owner of, and had regular access to, an email address
- Had regular access to the internet via mobile data plan and/or Wi-Fi

- Had stable housing and had remained at the same residence for at least 12 weeks prior to screening, with no anticipated housing changes during the duration of the study
- Understood how to use CT-155 beta during the screening visit, as assessed by the investigator during in-clinic installation and activation activities

Exclusion Criteria

A participant was not eligible for study entry if any of the following exclusion criteria were met:

- Was concurrently being treated with more than 2 antipsychotic medications (including more than 2 dosage forms)
- Was concurrently being treated with clozapine or haloperidol
- Had active prominent positive symptoms that, in the opinion of the investigator, would preclude effective engagement in treatment for negative symptoms
- Was concurrently receiving or had received psychotherapy within 12 weeks prior to screening
- Met either the International Classification of Diseases or DSM-5 criteria for diagnoses not under investigation, including schizophreniform, schizoaffective, or psychosis non-specific disorders
- Had post-traumatic stress disorder, bipolar disorder, major depressive disorder, developmental disorders, or any prominent disorder that would interfere with compliance to the protocol, per investigator judgment
- Had substance or alcohol use disorder (excluding caffeine and nicotine), that would interfere with compliance to the protocol, per investigator judgment
- Needed or was likely to require prohibited concomitant medications and/or therapy during the study, as determined by the investigator

- Was participating in another clinical study (interventional or observational) involving investigational drugs or devices
- Participated in the CT-155-C-001 clinical study
- Had suicidal ideation or behavior, as assessed by the Columbia-Suicide Severity Rating Scale (C-SSRS):
    - a. Participants who had a “yes” response to either Items 4 or 5 on the C-SSRS Suicidal Ideation Item within the last 12 weeks prior to screening or at baseline visit
    - b. Participants who had a “yes” response on the C-SSRS Suicidal Behavior Items within the last 26 weeks prior to screening or at baseline visit
    - c. Participants who, in the opinion of the investigator, presented a serious risk of suicide
- Showed any evidence of a clinically significant concomitant disease or any other clinical condition that would jeopardize participant safety while in the clinical study, as judged by the investigator

Supplementary tables

Supplementary Table 1: Concomitant antipsychotics, dose range, and route of administration

	N	Route of administration	Dose, range, mg
**Diazepines, oxazepines, thiazepines, and oxepines**	**31**		
Olanzapine	14	Oral	10–30
Quetiapine	3	Oral	100–450
Quetiapine fumarate	14	Oral	50–800
**Other antipsychotics**	**18**		
Aripiprazole	1	Intramuscular	400
Aripiprazole	9	Oral	5–30
Aripiprazole lauroxil	1	Intramuscular	882
Paliperidone palmitate	1	Intramuscular	39
Risperidone	6	Oral	0.5–4
**Indole derivatives**	**7**		
Lurasidone	1	Oral	120
Lurasidone hydrochloride	5	Oral	10–60
Ziprasidone	1	Oral	80

At baseline, concomitant use of second-generation antipsychotics was reported by 50 participants (n=44 receiving 1 antipsychotic; n=6 receiving 2 antipsychotics)

Supplementary Table 2: Correlations analysis between baseline CAINS-MAP scores and CT-155 beta engagement metrics during the 7-week intervention period (N=50)

	Number of days CT-155 beta was opened	Number of sessions	Number of therapeutic lessons completed	Number of goal-centered activities completed per participant	Total number of goals completed per participant
**Baseline CAINS-MAP score, Spearman's coefficient (95% CI)**	-0.11 (-0.38, 0.17)	-0.17 (-0.43, 0.12)	-0.12 (-0.39, 0.16)	-0.10 (-0.37, 0.18)	-0.11 (-0.38, 0.17)

CAINS-MAP, clinical assessment interview for negative symptoms, motivation and pleasure subscale; CI, confidence interval.

Supplementary Table 3: Modified[a] MARS domain and app-specific item scores at Week 7

	Score, mean (SD) n=43[b]
**Domain**	
Engagement	3.8 (0.8)
Functionality	4.2 (0.8)
Esthetics	3.9 (0.8)
Information quality[a]	4.0 (0.9)
Subjective quality	3.7 (0.8)
**App-specific items**	
Awareness: This app is likely to increase awareness of the importance of addressing negative symptoms	3.9 (1.3)
Knowledge: This app is likely to increase knowledge/understanding of negative symptoms	4.2 (1.1)
Attitudes: This app is likely to change attitudes toward improving negative symptoms	4.0 (1.2)
Intention to change: This app is likely to increase intentions/motivation to address negative symptoms	3.7 (1.2)
Help seeking: Use of this app is likely to encourage further help seeking for negative symptoms	3.8 (1.2)
Behavior change: Use of this app is likely to decrease negative symptoms	3.7 (1.4)

[a]Two items were omitted from the information subscale due to lack of relevance; [b]Data presented from participants with observed data (n=43).

MARS includes several domains: Engagement (items 1–5), functionality (items 6–9), esthetics (items 10–12), and information quality (items 14–18). Items were rated as either 1 (inadequate), 2 (poor), 3 (acceptable), 4 (good), or 5 (excellent) and statistically summarized per domain. The MARS also includes a subjective quality domain (items 20–23), which is scored separately using a 5-point Likert scale, with higher scores indicating better quality. The MARS app-specific items were scored as either 1 (strongly disagree), 2 (slightly disagree), 3 (neutral), 4 (slightly agree), or 5 (strongly agree).

MARS, mobile application rating scale; SD, standard deviation.

Supplementary Table 4: Screening and baseline assessments

Name of assessment	Description	Timepoint assessment was conducted
Motivation and Pleasure – Self Report[55]	A validated self-report tool that measures the motivation and pleasure domains of negative symptoms, derived from the CAINS-MAP, where lower scores indicate more severe symptom severity	Screening
Columbia-Suicide Severity Rating Scale[67]	A validated assessment of suicidal ideation and behavior (see exclusion criteria in **supplementary material** for further details)	Screening
Mobile Device Proficiency Questionnaire[68]	A validated scale designed to measure mobile device proficiency and digital literacy in older adults where higher scores indicate greater ability	Baseline
Clinical Assessment Interview for Negative Symptoms – Motivation and Pleasure[8]	A validated scale designed to assess the avolition, asociality, anhedonia domains of negative symptoms, where higher scores indicate more severe symptoms (see **Supplementary Table 3** for more details)	Baseline
Brief Assessment of Cognition in Schizophrenia[69]	A validated assessment of cognition that includes 3 tasks: symbol coding task assessing working memory, verbal memory task and digit sequencing task, assessing attention, and processing speed of information	Baseline
Defeatist Beliefs subscale[70] of the Dysfunctional Attitudes Scale[71]	An assessment of defeatist beliefs, where higher scores indicate more severe defeatist thinking	Baseline
Personal and Social Performance scale[72]	A validated clinician-rated scale that measures personal and social functioning in 4 domains: socially useful activities, personal and social relationships, self-care, and disturbing and aggressive	Baseline

	behaviors, where lower score indicate greater disability	

Supplementary Table 5: CAINS subscales[8]

CAINS Subscale	Symptom domain	CAINS item
Expression	Blunted affect, alogia	Vocal prosody<br>Gestures<br>Facial<br>Speech
Motivation and Pleasure	Avolition, asociality, anhedonia	Recreation<br>Expected pleasure<br>Past-week pleasure<br>Motivation<br>Social<br>Expected pleasure<br>Past-week pleasure<br>Friendships<br>Family relationships<br>Motivation<br>Vocational<br>Expected pleasure<br>Motivation

CAINS, clinical assessment interview for negative symptoms.

Supplementary Table 6: MARS domains[65]

Domain and description	Items
**Engagement**: Fun, interesting, customizable, interactive, well-targeted to audience	1. Entertainment: Is the app fun/entertaining to use? Does it use any strategies to increase engagement through entertainment (e.g., through gamification)?<br>2. Interest: Is the app interesting to use? Does it use any strategies to increase engagement by presenting its content in an interesting way?<br>3. Customization: Does it provide/retain all necessary settings/preferences for apps features (e.g. sound, content, notifications, etc.)?<br>4. Interactivity: Does it allow user input, provide feedback, contain prompts (reminders, sharing options, notifications, etc.)?<br>5. Target group: Is the app content (visual information, language, design) appropriate for your target audience?
**Functionality**: App functioning, easy to learn, navigation, flow logic, and gestural design of app	6. Performance: How accurately/fast do the app features (functions) and components (buttons/menus) work?<br>7. Ease of use: How easy is it to learn how to use the app; how clear are the menu labels/icons and instructions?<br>8. Navigation: Is moving between screens logical/accurate/appropriate/ uninterrupted; are all necessary screen links present?<br>9. Gestural design: Are interactions (taps/swipes/pinches/scrolls) consistent and intuitive across all components/screens?
**Esthetics**: Graphic design, overall visual appeal, color scheme, and stylistic consistency	10. Layout: Is arrangement and size of buttons/icons/menus/content on the screen appropriate or zoomable if needed?<br>11. Graphics: How high is the quality/resolution of graphics used for buttons/icons/menus/content?<br>12. Visual appeal: How good does the app look?
**Information quality**: Contains high quality information (e.g. text, feedback, measures, references) from a credible source	13. Accuracy of app description (in app store): Does app contain what is described?[a]<br>14. Goals: Does app have specific, measurable, and achievable goals (specified in app store description or within the app itself)?<br>15. Quality of information: Is app content correct, well written, and relevant to the goal/topic of the app?<br>16. Quantity of information: Is the extent coverage within the scope of the app; and comprehensive but concise?<br>17. Visual information: Is visual explanation of concepts – through charts/graphs/images/videos, etc. – clear, logical, correct?

	18. Credibility: Does the app come from a legitimate source (specified in app store description or within the app itself)?<br>19. Evidence base: Has the app been trialed/tested; must be verified by evidence (in published scientific literature)?[a]
**Subjective quality**	20. Would you recommend this app to people who might benefit from it?<br>21. How many times do you think you would use this app in the next 12 months if it was relevant to you?<br>22. Would you pay for this app?<br>23. What is your overall star rating of the app?
**App-specific items**	24. Awareness: This app is likely to increase awareness of the importance of addressing negative<br>25. Knowledge: This app is likely to increase knowledge/understanding of negative symptoms<br>26. Attitudes: This app is likely to change attitudes toward improving negative symptoms<br>27. Intention to change: This app is likely to increase intentions/motivation to address negative symptoms<br>28. Help seeking: Use of this app is likely to encourage further help seeking for negative symptoms<br>29. Behavior change: Use of this app is likely to decrease negative symptoms

[a]These items were excluded during this study, due to lack of relevance.

Items are rated as either 1 (inadequate), 2 (poor), 3 (acceptable), 4 (good), or 5 (excellent) and statistically summarized per domain. The MARS subjective quality domain (items 20–23) is scored separately using a 5-point Likert scale, with higher scores indicating better quality. The app specific items were scored as either 1 (strongly disagree), 2 (slightly disagree), 3 (neutral), 4 (slightly agree), or 5 (strongly agree).

MARS, mobile application rating scale.

Supplementary figures

Supplementary Figure 1: Time to final engagement (session 60 seconds or longer in duration) with CT-155 beta

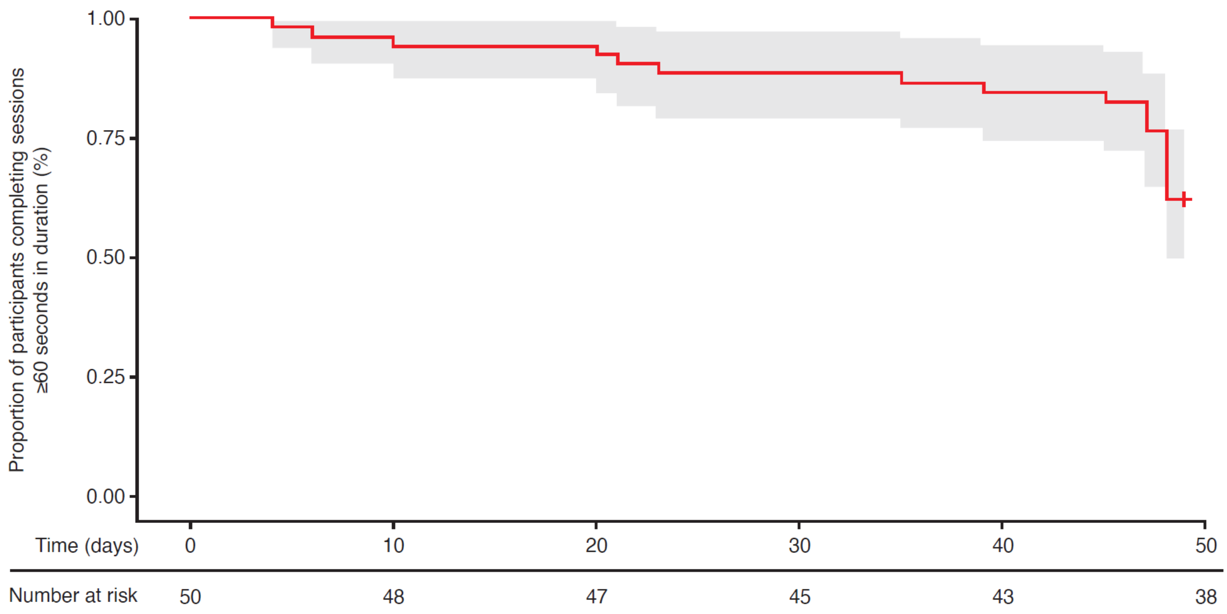

The sharp drop in engagement over the last couple of study days is explained by the fact that as there would be insufficient time to complete certain goals the app provided users shorter duration tasks therefore not meeting the ≥60 second threshold.

Supplementary Figure 2: MARS app-specific item scores at Week 7

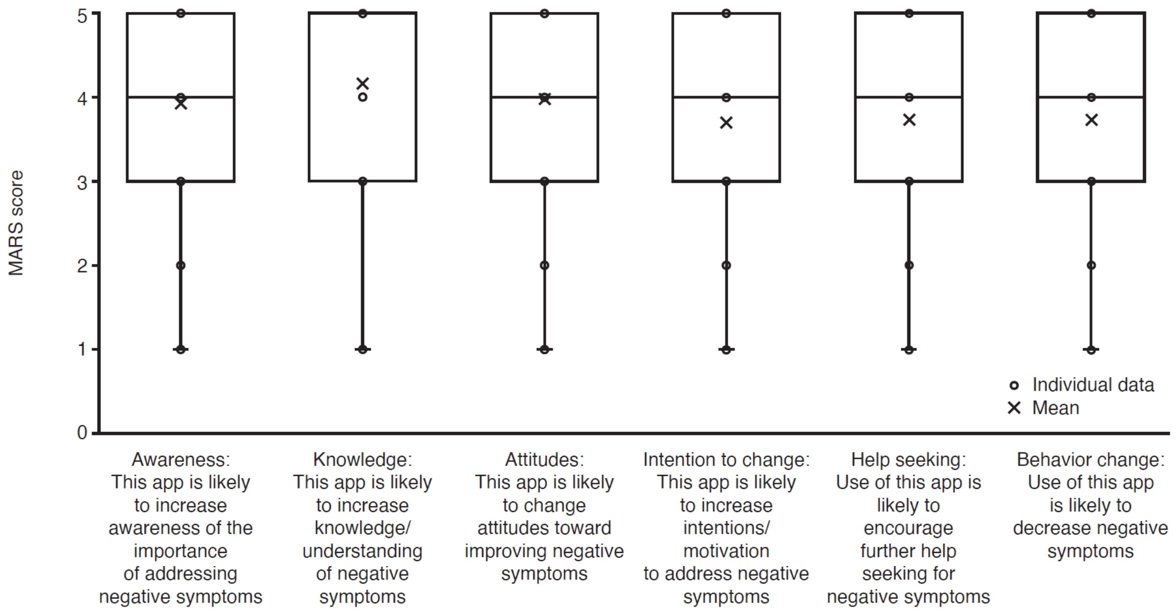

Data presented from participants with observed data (n=43). Box plots depict the median and interquartile range of participant engagement metrics; the median is represented by a horizontal line in each box. The whiskers depict range within 1.5 times the interquartile range (IQR), values outside this range are considered outliers

The MARS app-specific items were scored as either 1 (strongly disagree), 2 (slightly disagree), 3 (neutral), 4 (slightly agree), or 5 (strongly agree).

MARS, Mobile App Rating Scale.
